# A Case of Pyelonephritis Caused by Ureteral Obstruction due to a Urinary Catheter

**DOI:** 10.1002/ccr3.71161

**Published:** 2025-10-06

**Authors:** Minaho Nonaka, Hiromu Naraba, Ichiro Hirayama, Tatsuhiko Hayashi, Tetsuhiro Yano, Mitsuru Ishii, Yoshiteru Tominaga

**Affiliations:** ^1^ Department of Emergency Medicine National Hospital Organization Saitama Hospital Saitama Japan; ^2^ Department of Clinical Toxicology, Faculty of Medicine Saitama Medical University Saitama Japan

**Keywords:** acute kidney injury, pyelonephritis, sepsis, ureteral obstruction, urinary tract infections

## Abstract

An 88‐year‐old man with long‐term catheterization developed sepsis and obstructive pyelonephritis due to catheter migration into the left ureter. After recovery with treatment, recurrence was revealed following routine exchange. Clinicians should suspect ureteral migration in elderly, bedridden patients with long‐term urinary catheters who present with peri‐catheter leakage or renal dysfunction.

**Summary**

- Migration of an indwelling urinary catheter into the ureter may cause urinary retention despite catheter placement.
- Sudden lateral urinary leakage should prompt suspicion of catheter misplacement.
- Early detection can prevent complications such as obstructive pyelonephritis and sepsis, potentially improving clinical outcomes.

## 1 Case Presentation

An 88‐year‐old male with an indwelling urinary catheter for benign prostatic hyperplasia was admitted with fever and altered consciousness. On arrival, vital signs suggested sepsis: temperature 39.2°C; BP 109/72 mmHg; HR 111 bpm; RR 16/min; SpO_2_ 94% on supplemental oxygen; Glasgow Coma Scale E3V4M6.

**FIGURE 1:**
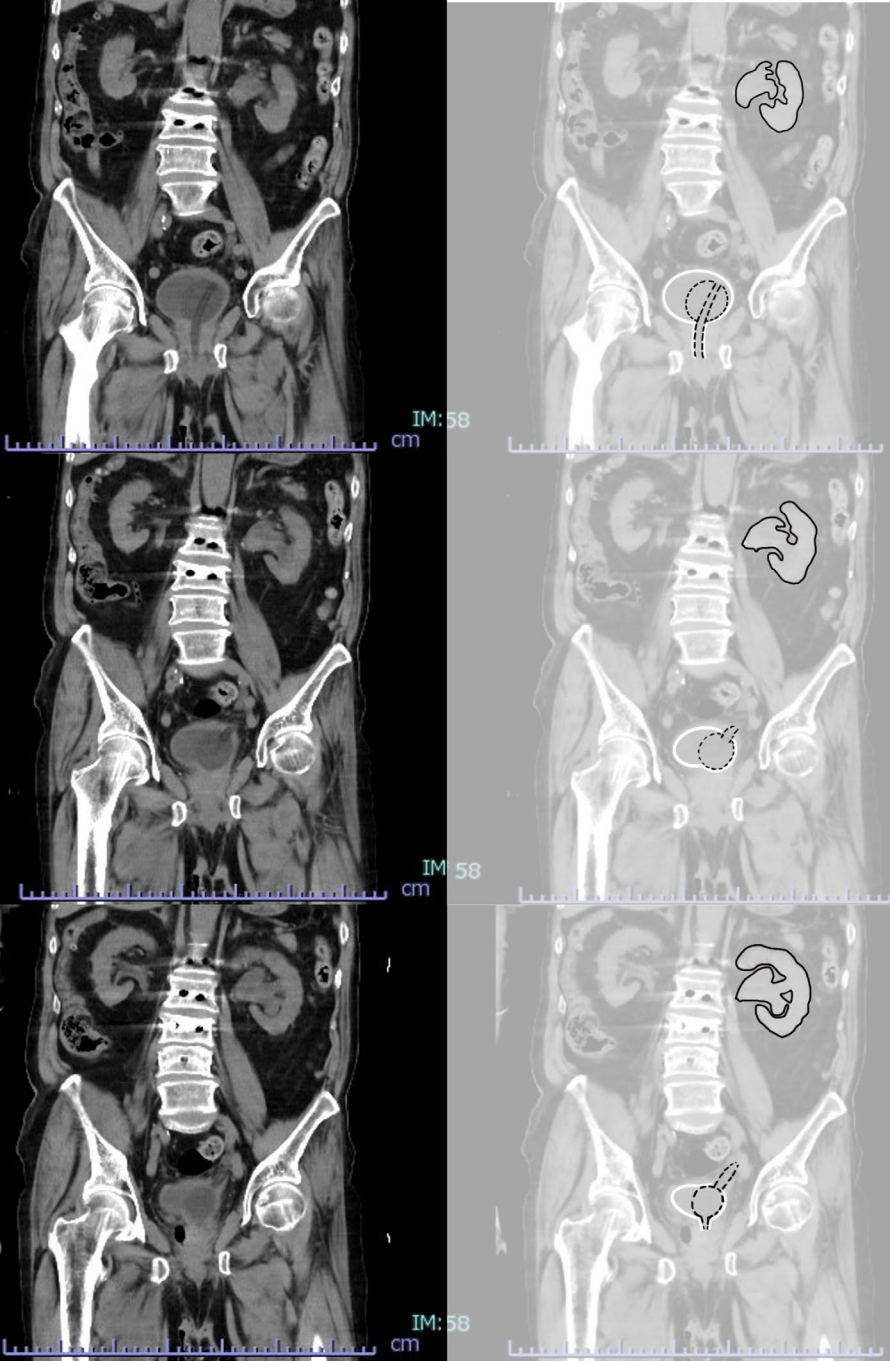
CT (coronal section, at the first stray of the urinary catheter). Black line: left kidney, renal pelvis. Black dotted line: urethral catheter. White line: bladder, urethra. The tip of the urethral catheter strayed into the left ureter, causing left hydronephrosis (SFU grade 3).

Laboratory results revealed severe renal dysfunction and systemic inflammation: BUN 159.0 mg/dL, Cr 6.98 mg/dL, WBC 8100/μL, and CRP 29.98 mg/dL. Urinalysis revealed pyuria and bacteriuria, and the SOFA score was 9. Non‐contrast abdominal CT showed the catheter tip had migrated into the left ureter (Figure 1), causing hydronephrosis (SFU grade 3), consistent with obstructive pyelonephritis.

**FIGURE 2:**
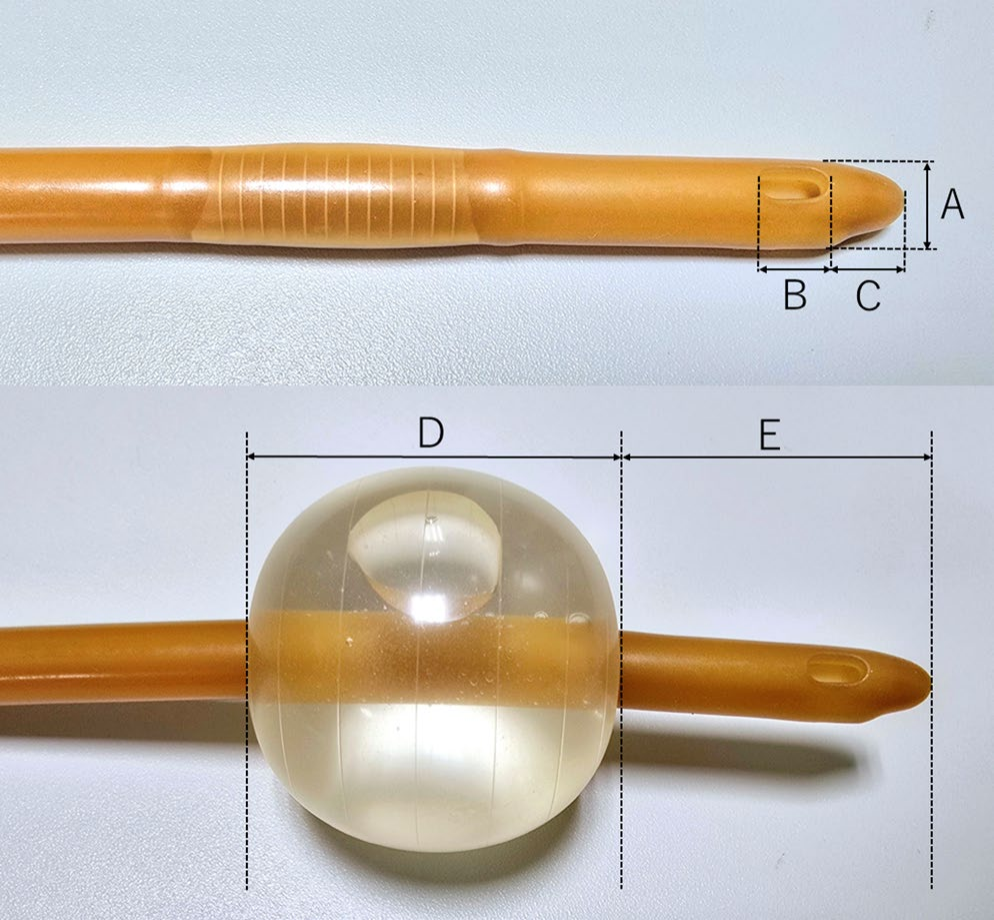
20 Fr, 3‐way, 30 mL Foley catheter. (A) Urinary catheter diameter 8 mm. (B) Length of side hole of the urinary catheter 6 mm. (C) Length from the tip of the urethral catheter to the side hole 8 mm. (D) Urinary catheter balloon diameter (after injecting 10 cc of distilled water) 45 mm. (E) Length from the tip of the urinary catheter to the balloon 30 mm.

A urologist replaced the catheter with a new 20 Fr, 3‐way, 30 mL Foley catheter (Figure 2). The patient had a history of multiple self‐removals of urinary catheters, so a 20 Fr, 3‐way, 30 mL Foley catheter was used routinely to prevent dislodgement by his attending outpatient urologist. Empirical tazobactam/piperacillin was started. MRSA was later identified, and teicoplanin was added. Sepsis resolved after 2 weeks, though discharge was delayed due to logistical reasons. One month later, the same catheter type was replaced during routine care. Three days later, urinary leakage from the external urethral orifice was noted. CT confirmed catheter migration into the left ureter, identical to the first episode. A 16 Fr, 2‐way, 10 mL Foley catheter was placed, and no recurrence has been observed since.

## 2 Discussion

This case underscores the importance of recognizing ureteral migration of an indwelling urethral catheter, as early detection may prevent serious complications such as hydronephrosis and urosepsis. Increased awareness of peri‐catheter urine leakage as a potential sign of catheter misplacement is essential for timely diagnosis and intervention, contributing to improved patient outcomes.

**FIGURE 3:**
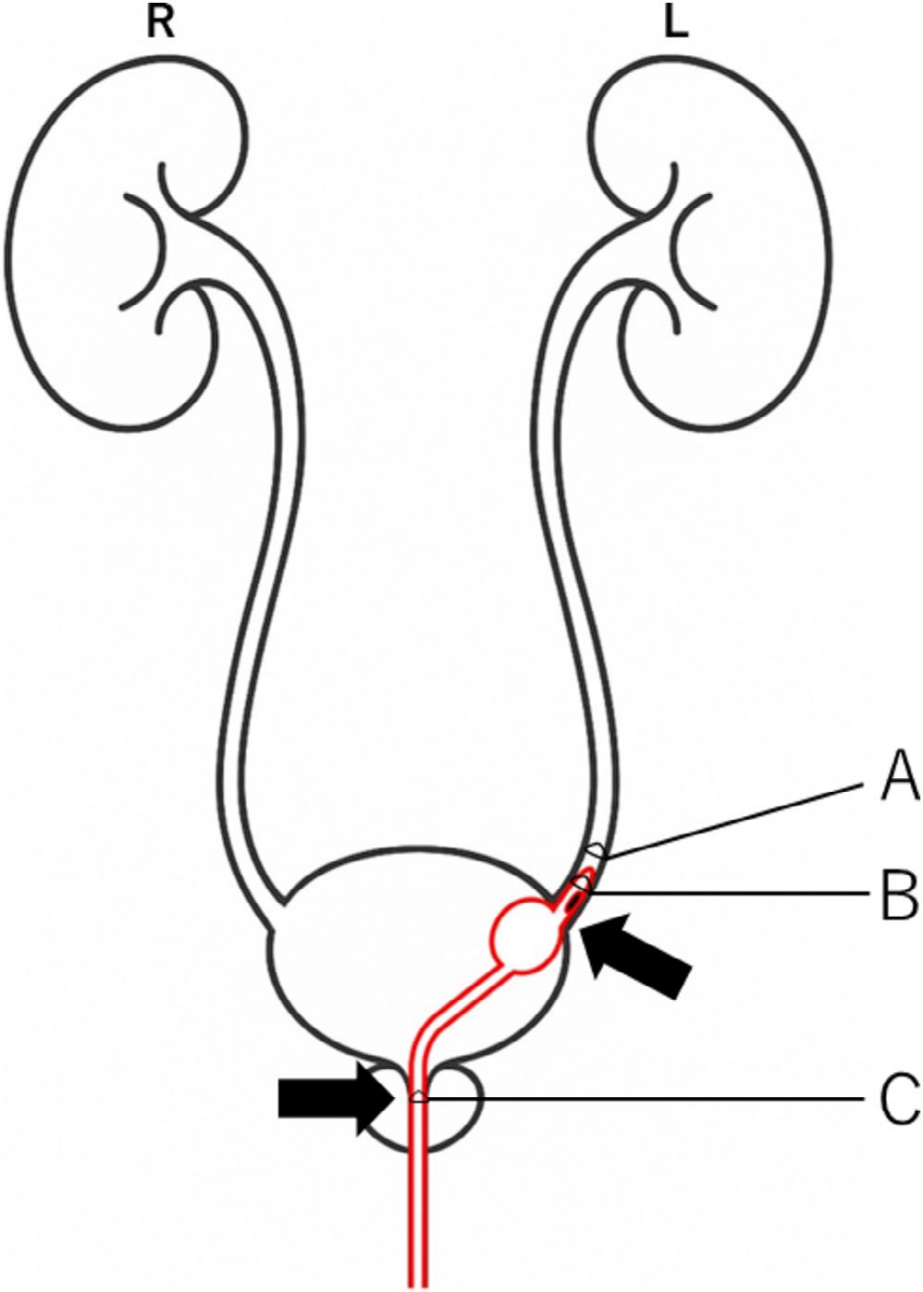
Schema of the patient's urethral catheter, ureter, bladder, and urethra. (A) Internal diameter of distal ureter 8.0 mm. (B) Urethral catheter diameter 8.0 mm. (C) Internal diameter of urethra 8.0 mm. The left ureter is obstructed because (A) and (B) are the same length and the urethral catheter is a side‐opening catheter. Because (C) and (B) are the same length, urine in the bladder is not excreted and urine leaks from the side of the urethral catheter when the pressure in the bladder increases.

Although rare, ureteral migration is a documented complication [1, 2, 3], typically presenting with abdominal pain or fever. As illustrated in Figure 3, if the tip of a laterally perforated catheter enters the ureter, it may obstruct urinary flow from the affected kidney. Simultaneously, urethral occupancy may block contralateral drainage, causing bilateral retention, and elevated bladder pressure. This may lead to leakage from the external urethral orifice and, if unrecognized, progress to hydronephrosis or sepsis.

To our knowledge, recurrent ureteral misplacement has not been previously reported. Although this patient experienced two episodes, no anatomical abnormalities were identified on CT, suggesting an incidental mechanism. A prior review [3] reported that 13 of 20 patients with catheter migration had long‐term catheterization; 6 were elderly (> 70 years old), and 3 had spinal cord injury. Thus, long‐term catheterization and immobility with reduced ADL may be risk factors.

In elderly, bedridden patients with long‐term catheters, peri‐catheter leakage should raise suspicion of obstruction or ureteral migration. Both can cause hydronephrosis or obstructive pyelonephritis and require prompt evaluation. While ultrasound may detect obstruction, CT is often necessary to confirm misplacement. If obstruction is suspected, catheter replacement and close monitoring of vital signs, urine output, and labs are recommended. In confirmed cases, adjusting catheter size or fixation and consulting a urologist may help prevent recurrence.

## Author Contributions

**Minaho Nonaka:** writing – original draft. **Hiromu Naraba:** writing – review and editing. **Ichiro Hirayama:** supervision. **Tatsuhiko Hayashi:** data curation. **Tetsuhiro Yano:** visualization. **Mitsuru Ishii:** conceptualization. **Yoshiteru Tominaga:** project administration.

## Ethics Statement

The authors have nothing to report.

## Consent

Written informed consent was obtained from the patient to publish this case report and its accompanying image.

## Conflicts of Interest

The authors declare no conflicts of interest.

## Data Availability

The data that support the findings of this study are available from the corresponding author upon reasonable request.
